# Team interaction behaviors correlates with team creativity among nursing students: Canonical correlation and moderation analyses

**DOI:** 10.1186/s12912-024-02158-7

**Published:** 2024-07-16

**Authors:** Hsing-Yuan Liu, Hui-Mei Han, Hsiu-Fang Chen, Chin-Yen Han, Ding-Hau Huang, Ding-Yang Hsu, Chen-Hung Chiang

**Affiliations:** 1 Department of Nursing, Chang Gung University of Science and Technology, Taoyuan City, Taiwan; 2 Department of Nursing, New Taipei Municipal TuCheng Hospital (Built and Operated By Chang Gung Medical Foundation), New Taipei City, Taiwan; 3 Department of Nursing, Chang Gung University, Taoyuan City, Taiwan; 4 Institute of Creative Design and Management, National Taipei University of Business, Taoyuan City, Taiwan; 5 Department of Industrial Design, Ming Chi University of Technology, New Taipei City, Taiwan; 6 Department of Gerontology and Health Care Management, Chang Gung University of Science and Technology, Taoyuan City, Taiwan

**Keywords:** Interaction behavior, Team creativity, Moderating effect, Nursing education, Nursing students

## Abstract

**Background:**

Prior studies have indicated team members’ interaction behaviors may predict creativity among nursing students.

**Methods:**

This study investigated the correlation between interaction behaviors and creativity, both individual- and team-level, among nursing students. In this cross-sectional quantitative study, data were obtained from self-reported questionnaires. Individual creativity was assessed using the Torrance Tests of Creative Thinking scale; the perceived team interaction behavior and team creativity were assessed using validated instruments. Canonical correlation analysis was conducted to determine the overall correlation between interaction behaviors, and creativity, and the moderating effect of female proportion dominance was also examined.

**Results:**

A total of 164 nursing students (84.1% female) arranged into 14 teams were included in this study. Canonical correlation analysis showed a positive correlation between interaction behaviors and creativity (correlation = 0.88). All dimensions of interactive behaviors were positively related to creativity dimensions. A stronger correlation to team creativity (correlation = 1) was found compared to individual creativity (correlation = 0.07). This study demonstrated that individual interactive behaviors including spontaneous communication and helping behavior predicted high team creativity.

**Conclusions:**

This insight may be valuable for nursing education programs seeking to foster creativity and effective teamwork. The potential moderating effect of female proportions on team interaction behaviors and creativity should be investigated further.

**Supplementary Information:**

The online version contains supplementary material available at 10.1186/s12912-024-02158-7.

## Introduction

As the global demand for personalized healthcare continues to rise, there has been a substantial escalation in the need for healthcare professionals to communicate and collaborate effectively. This is particularly crucial for providing interdisciplinary patient care and addressing complex healthcare challenges [1]. Studies have also indicated that nursing students who learn to work collaboratively within healthcare teams tend to exhibit improved nursing practice and enhanced problem-solving abilities [2, 3]. In response to these evolving requirements, nursing training curricula are being revised with more emphasis on fostering teamwork and collaboration. These curricular adaptations are designed to prepare nurses to meet the demands of the dynamic healthcare environment [4, 5].

A growing body of evidence has indicated that a significant portion of creative behaviors is most evident in team contexts. Interdisciplinary education programs with a particular emphasis on interactive behaviors have been shown to foster team creativity [6]. Several interactive behaviors have been identified to enhance teamwork competency including constructive controversy, helping behavior, and spontaneous communication [6–8]. Consequently, enhancing interaction behaviors that contribute positively to creativity and innovation—characterized by the implementation of creative ideas within the teams, has become a key element in addressing complex healthcare challenges faced by healthcare teams [7].

In addition to the established positive factors of team interaction behaviors, such as cooperation, collaboration, and communication among team members [9], there is a range of other variables and mediators that may also influence team interactions. For instance, the potential impact of demographic variation on team effectiveness and the influence of gender composition on team interactions and outcomes have been the subject of investigation, but findings have been inconsistent. Some suggested that a team's demographic composition can influence the degree to which group members display helping behaviors [10]. In contrast, some researchers found no significant relationship between gender composition and constructive controversy [11]. The influence of gender diversity on team creativity has also yielded inconclusive findings. Some studies have reported that gender diversity fosters team creativity or innovation [12–15]. Conversely, gender diversity has been associated with negative impacts [16], or found to have no significant effects on team creativity [17, 18]. In the context of the nursing profession, which is a female-dominant occupation, the impact of more homogenous gender composition on team interaction behavior and team creativity has not been explored in detail.

While existing research has explored team creativity from either an individual perspective [19, 20] or a team-based viewpoint [21, 22], only a limited number of studies have comprehensively examined team creativity from both angles. A prior study conducted within a telecom company suggested that team creativity is influenced by individual creativity and shared mental models [23]. As for nursing education, previous studies have predominantly focused on individual creativity and pedagogical approaches that promote collaborative teamwork, and much less is known about how team interactions and team creativity impact collaborative outcomes within nursing teams.

This study aimed to investigate positive team interactive behaviors that correlate with creativity, both at individual and team levels in nursing teams. We proposed that the dynamics of team interaction played a pivotal role in team creativity, and that team interaction behaviors would correlate with the creativity of individual team members as well as team creativity. Considering that nursing is typically a female-dominant occupation, this study also attempted to examine the potential moderating effects of female-dominant teams on the relationships between team interaction behaviors and creativity.

This enhanced understanding of the association between team interactive behaviors and creativity may inform curriculum aimed at promoting effective collaboration and enhancing creativity in nursing teams, ultimately contributing to improved healthcare practices.

## Methods

### Research design

This is a cross-sectional, survey study. Data were collected from self-reported surveys of nursing students.

### Sample and setting

Nursing students from a science and technology university in northern Taiwan who were enrolled in an interdisciplinary course as part of their 2- or 4-year nursing programs were included in this study. After signing informed consent forms, participants received a coded package with the relevant questionnaires. Approval was obtained from the Institutional Review Board (IRB) of the hospital ethics committees (IRB number: 201800212B0) before data collection. G*Power was used to determine the minimum sample size of 77 (setting was: predictor variables, 3; confidence interval: 95%; power: 0.81).

### Instruments

Three self-report questionnaires were scaled instruments asking for participants to rate their team interaction behaviors (TIB), team creativity (TCr) and individual creativity. Appendix shows sample statements from three instruments, each described and measured as follows:

#### Interaction behaviors

Team interaction behaviors were assessed using a validated 24-item questionnaire, translated and developed for the Chinese population [24]. This questionnaire has been used in several recent studies, the range of reliability was between 0.89 and 0.92 [8, 25]. A five-point Likert scale, from 1 (strongly disagree) to 5 (strongly agree), was used to rate survey questions. The questionnaire included subscales measuring spontaneous communication (10 questions), helpful behaviors (10 questions), and constructive controversy (4 questions). The three subscales were added to form the total scale score, and higher scores represented a greater perception of interaction behaviors. In this study, the three interaction behaviors have Cronbach's alpha coefficients ranging between 0.75 and 0.93.

#### Individual creativity

Individual creativity was assessed using the previously developed Torrance Tests of Creative Thinking (TTCT) – Taiwan version [26]. In brief, the TTCT consists of a verbal version (TTCT-V), and a figural version (TTCT-F). TTCT-V assesses three key constructs: fluency, flexibility, and originality, with a total score ranging from 0 to 176, and subscale scores for fluency, flexibility, and originality ranging from 0 to 50, 0 to 26, and 0 to 100. TTCT-F includes the same constructs in TTCT-V, plus *elaboration* (measuring the amount of detail in the responses). TTCT-F's total score ranges from 0 to 377, with subscale scores for fluency, flexibility, originality, and elaboration ranging from 0 to 57, 0 to 35, 0 to 114, and 0 to 171, respectively. The Cronbach’s alpha coefficients were 0.84 for TTCT-V and 0.76 for TTCT-F for this study.

According to the recommendation by Okuda et al., 1991 [27], TTCT-V and TTCT-F could be regarded as two distinct forms of creativity. Therefore, in this study individual creativity was defined as the sum of TCT-V and TTCT-F. The Cronbach's alpha coefficient for individual creativity was 0.83, and validity for TTCT-V and TTCT-F were confirmed satisfactory using factor analyses.

#### Team creativity

Team creativity was assessed using a 10-item questionnaire [20], adapted from an instrument initially designed for the Chinese population [24]. Respondents rated all questions on a five-point Likert scale (1 = strongly disagree, 5 = strongly agree). The total score is defined as the average of the sum of scores for each item in the questionnaire. It has consisted high reported Cronbach’s alpha coefficient ranging from 0.86 to 0.95 [28–30], and the alpha reliability was 0.96 for this study.

### Data analysis

Age and gender proportions were described using mean ± standard deviation. Canonical correlation analysis was performed to determine the overall correlation between the two sets of variables— interaction behaviors and creativity. It is assumed in the canonical correlation analysis that the relationships should be linear i.e. assuming that there should be low multicollinearity in the data. If the two sets of data are highly inter-correlated, then the coefficient of the canonical correlation is invalid. The Kolmogorov–Smirnov test can be used for checking the assumption of the normality. If the test is significant (i.e. *p* value < 0.05) means that the multivariate normality is invalid. Structure coefficient (canonical loadings) > 0.45 was defined as indicative of a significant contribution to the canonical function, suggesting a higher probability of replicability [31, 32].

After identifying the relevant independent and dependent variables, we used the SPSS PROCESS macro [33], to investigate the potential moderating effects of the female proportion in the team on the relationships between team creativity (dependent variable) and each of the team interaction behaviors (independent variables). This analysis encompassed three models, where we performed regression analyses for students' team creativity scores, considering the proportion of females and three different interaction behaviors. Evaluation of model assumptions for the multiple regression analysis included linearity, multivariate normality, homoscedasticity, and independence (i.e. No multicollinearity). Scatterplots were employed to test whether there was a linear or curvilinear relationship. Multivariate normality assumed that the residuals of the multiple regression were normally distributed. Homoscedasticity states that the variance of error terms was similar across the values of the independent variables. A plot of standardized residuals versus predicted values was used to test whether points were equally distributed across all values of the independent variables. Finally, the multiple regression model assumed that the independent variables were not highly correlated with each other. Variance Inflation Factor (VIF) values were used to test for multicollinearity. Each model examined the interaction between the proportion of females and the impact of a particular interaction behavior on team creativity: This involved examining constructive controversy in Model 1, helping behaviors in Model 2, and spontaneous communication in Model 3. To avoid multicollinearity, mean centering was performed before conducting the multiple regression analyses to assess moderating effects. All analysis was conducted using SPSS version 20.0.

### Data aggregation

In this study, data aggregation was conducted at the team level, whereby the individual responses of team members to assessment instruments were combined to generate team-level scores for both perceived interaction behaviors and team creativity. The appropriateness of data aggregation was validated using within-group agreement (Rwg) [34]. Adequate aggregation of individual data to obtain team-level responses was considered when the Rwg value was ≥ 0.70 [35, 36]. In this study, the median Rwg for both interaction behaviors and team creativity was 0.99, indicating strong agreement among individual team members' responses. This observation affirmed the appropriateness of aggregating individual data to obtain team-level responses.

## Results

Among the 164 nursing students in this study, a majority of 138 (84.1%) were female, while 26 (15.9%) were male. The students had a mean age of 21.5 ± 0.75 years old. They were divided into 14 teams with ten teams comprised of 12 members and four teams of 11 members. The mean score for interaction behaviors construct were: helping behaviors (4.19, *SD* = 0.59), followed by spontaneous communication (4.07, *SD* = 0.73), and constructive controversy (4.06, *SD* = 0.59). The overall team creativity score was 4.16 (*SD* = 0.67). Every team in the study had a female-dominant gender composition, with proportions of female ranging from 75% to 91.7%. Teams 2, 5, 8, and 10 included 25% male students. It's worth noting that Team 8 had the lowest total mean scores for both the team interaction behaviors scale and team creativity. Detailed information regarding the gender composition, interaction behavior scores, and team creativity scores for each team can be found in Table 1.

**Table 1:** Demographics of student teams and the descriptive statistics of team interaction behavior and team creativity scales for each team (*n* = 14, *N* = 164)

				**Interaction Behaviors**					**Team Creativity**
**Team**	**Age**	**Gender, n (%)**			**Total score**	**CC**	**HB**	**SC**	
	**Years, M (SD)**	**Male**	**Female**		**M (SD)**	**M (SD)**	**M (SD)**	**M (SD)**	**M (SD)**
1	21.33 (0.49)	2 (16.7)	10 (83.3)		3.94 (0.87)	4.04 (0.84)	4.08 (0.84)	3.74 (1.01)	3.94 (0.87)
2	21.42 (0.67)	3 (25.0)	9 (75.0)		4.40 (0.59)	4.41 (0.53)	4.40 (0.56)	4.40 (0.73)	4.40 (0.59)
3	22 (1.65)	1 (8.3)	11 (91.7)		4.35 (0.52)	4.43 (0.37)	4.41 (0.41)	4.24 (0.53)	4.35 (0.42)
4	22.33 (0.49)	2 (16.7)	10 (83.3)		4.02 (0.58)	4.17 (0.62)	4.03 (0.68)	3.90 (0.55)	4.02 (0.58)
5	21.83 (0.94)	3 (25.0)	9 (75.0)		4.14 (0.39)	4.10 (0.46)	4.18 (0.46)	4.13 (0.47)	4.14 (0.39)
6	21.42 (0.51)	2 (16.7)	10 (83.3)		4.29 (0.73)	4.15 (0.69)	4.37 (0.79)	4.32 (0.77)	4.29 (0.73)
7	21.50 (0.52)	1 (8.3)	11 (91.7)		4.10 (0.44)	4.01 (0.54)	4.19 (0.50)	4.08 (0.44)	4.10 (0.44)
8	21.25 (0.45)	3 (25.0)	9 (75.0)		3.28 (1.25)	3.30 (0.71)	3.71 (0.77)	3.58 (0.77)	3.56 (0.71)
9	21.50 (0.67)	1 (8.3)	11 (91.7)		4.12 (0.39)	3.64 (0.38)	4.29 (0.46)	4.28 (0.46)	4.12 (0.39)
10	21.42 (0.51)	3 (25.0)	9 (75.0)		3.88 (0.56)	3.86 (0.60)	3.94 (0.72)	3.83 (0.67)	3.89 (0.66)
11	21.36 (0.50)	2 (16.7)	10 (83.3)		3.72 (1.05)	3.82 (1.10)	3.75 (1.12)	3.62 (1.05)	3.72 (1.05)
12	21.27 (0.47)	1 (9.1)	10 (90.9)		4.04 (1.01)	4.03 (0.45)	4.44 (0.43)	4.40 (0.49)	4.04 (1.02)
13	21.18 (0.40)	1 (9.1)	10 (90.9)		3.86 (1.07)	4.21 (0.50)	3.77 (1.32)	3.80 (1.20)	3.86 (1.07)
14	21.55 (0.52	1 (9.1)	10 (90.9)		4.11 (1.05)	4.69 (0.61)	4.51 (0.45)	4.34 (0.57)	4.11 (1.05)

*CC* Constructive controversy, *HB* Helping behaviors, *SC* Spontaneous communication

The overall mean total score for individual creativity was 90.57 (SD = 31.39), which comprised of the TTCT-V score (mean: 44.04 (SD = 24.70)) and the TTCT-F score (mean: 46.53 (SD = 17.13)). Detailed TTCT-V and TTCT-F scores for each team can be found in Table 2. Notably, Team 8, which had the lowest team creativity score had the highest individual creativity score for scale TTCT-V and TTCT-F.

**Table 2:** Individual creativity measured by Torrance Tests of Creative Thinking (TTCT): Verbal (V) and Figural (F) for each team (*n* = 14, *N* = 164)

**Team**	**TTCT-V**				**TTCT-F**					
	**Total Score**	**FLUE**	**FLEX**	**ORIG**	**Total Score**	**FLUE**	**FLEX**	**ORIG**	**ELAB**	**TTCT**
	**M (SD)**	**M (SD)**	**M (SD)**	**M (SD)**	**M (SD)**	**M (SD)**	**M (SD)**	**M (SD)**	**M (SD)**	**M (SD)**
1	23.17 (10.07)	9.67 (4.52)	6.42 (2.23)	7.08 (4.10)	35.42 (10.60)	15.25 (6.66)	8.67 (3.23)	9.25 (6.03)	2.25 (1.60)	58.58 (22.60)
2	33.42 (12.58)	15.25 (5.77)	8.33 (2.19)	9.83 (6.01)	32.50 (14.51)	14.25 (6.59)	8.58 (2.68)	7.42 (5.21)	2.25 (1.42)	65.92 (18.32)
3	16.50 (5.76)	7.25 (2.34)	5.92 (1.68)	3.33 (2.53)	36.83 (15.32)	15.25 (6.51)	9.33 (2.64)	9.67 (6.14)	2.58 (1.98)	53.33 (17.94)
4	39.75 (15.53)	19.00 (8.03)	9.33 (2.42)	11.42 (6.24)	53.00 (13.06)	23.92 (4.60)	12.33 (2.40)	14.92 (6.88)	1.83 (1.19)	92.75 (24.95)
5	43.42 (17.02)	20.00 (7.64)	10.67 (2.39)	12.75 (8.94)	53.67 (10.08)	22.50 (5.33)	13.59 (3.45)	16.08 (3.55)	1.50 (1.24)	97.08 (20.97)
6	38.67 (16.25)	18.17 (7.25)	8.92 (3.12)	11.58 (2.39)	47.92 (9.53)	22.00 (4.45)	10.67 (1.72)	13.17 (6.38)	2.08 (1.56)	86.58 (21.90)
7	47.92 (21.13)	21.75 (8.45)	9.17 (1.99)	17.00 (11.76)	48.33 (12.32)	24.25 (6.48)	10.83 (2.44)	11.75 (7.86)	1.50 (0.67)	96.25 (22.48)
8	66.67 (23.54)	30.08 (10.65)	12.08 (1.31)	24.50 (12.49)	59.25 (16.58)	27.42 (7.73)	11.92 (2.27)	18.33 (8.47)	1.58 (0.67)	125.92 (33.16)
9	60.08 (29.41)	27.00 (13.00)	9.83 (2.41)	23.25 (14.92)	57.50 (16.22)	25.16 (6.86)	10.92 (1.88)	15.50 (7.83)	1.92 (0.90)	113.58 (34.42)
10	32.92 (22.34)	14.75 (7.58)	7.50 (3.63)	10.67 (9.12)	38.33 (14.76)	16.50 (7.12)	10.17 (3.46)	10.08 (4.42)	1.58 (1.24)	71.25 (35.09)
11	24.86 (11.53)	10.80 (5.44)	7.22 (2.64)	5.93 (4.24)	37.40 (19.08)	16.93 (9.05)	8.54 (3.22)	9.70 (7.84)	2.35 (2.45)	62.09 (28.13)
12	40.53 (22.01)	17.20 (8.91)	8.97 (3.94)	14.52 (10.00)	49.84 (24.63)	19.92 (9.47)	11.52 (4.40)	15.90 (8/90)	2.79 (2.37)	90.26 (45.15)
13	35.67 (20.15)	16.08 (8.87)	8.49 (2.59)	11.17 (9.75)	49.05 (20.26)	19.37 (6.60)	12.20 (4.13)	14.99 (10.47)	2.53 (1.50)	84.60 (38.91)
14	28.51 (10.61)	12.41 (5.13)	7.72 (3.05)	8.48 (4.33)	42.77 (18.15)	17.55 (8.74)	10.09 (2.91)	13.54 (7.65)	1.79 (1.47)	71.16 (24.61)

*FLUE* Fluency, *FLEX* Flexibility, *ORIG* Originality, *ELAB* Elaboration

### Canonical Correlation Analysis

Table 3 presented the results of the canonical correlation analysis, showing one significant function with squared canonical correlations (R_c_^2^) of 0.77 (Table 3). The overall significance of the model was demonstrated through multivariate tests (Wilk’s λ representing unexplained variance in the model = -0.24, *F* (3, 160) = 55.44, *p* < 0.001). These results confirmed that the model accounted for a substantial proportion (76.1%) of the shared variance between the sets of variables. The findings imply strong relationships between each interaction behavior and the team creativity constructs. Redundancy analysis was performed to further explore the extent to which the canonical function explains the variance between the interaction behaviors and creativity sets, and the outcome is presented in Table 3.

**Table 3:** Canonical correlation between total perceived interaction behaviors and creativity (individual and team creativity) scores (*N* = 164)

Variables	Canonical Function I			Interaction behaviors	Creativity
	**Coeff**	**Loading (r**_**s**_**)**	**r**_**s**_^**2**^** (%)**	**Explained Variance (%)**	**Explained Variance (%)**
TC	1.00	1.00^a^	99.80		
IC	-0.20	0.07	0.52		
R_c_^2^	0.77				
				81.59	25.57
CC	0.16	0.79^a^	62.25		
HB	0.34	0.94^a^	88.74		
SC	0.57	0.97^a^	93.90		
				62.41	33.43

*CC* Constructive Controversy, *HB* Helping Behaviors, *SC* Spontaneous Communication, *TC* Team Creativity, *IC* Individual Creativity, Coeff. Standardized canonical function coefficient, *r*_*s*_ Structure coefficient, *r*_*s*_^*2*^ Squared structure coefficient, *R*_*c*_^*2*^ Squared canonical correlations

^a^r_s_ > .45

The set of independent variables (interaction behaviors) accounted for 62.41% of the variance across the three interaction behavior constructs and 81.59% of the variability within the two creativity constructs. In contrast, the set of dependent variables (creativity) elucidated 25.57% of the variability within the two creativity constructs and 33.43% of the variability within the three interaction behavior constructs.

**Fig. 1:**
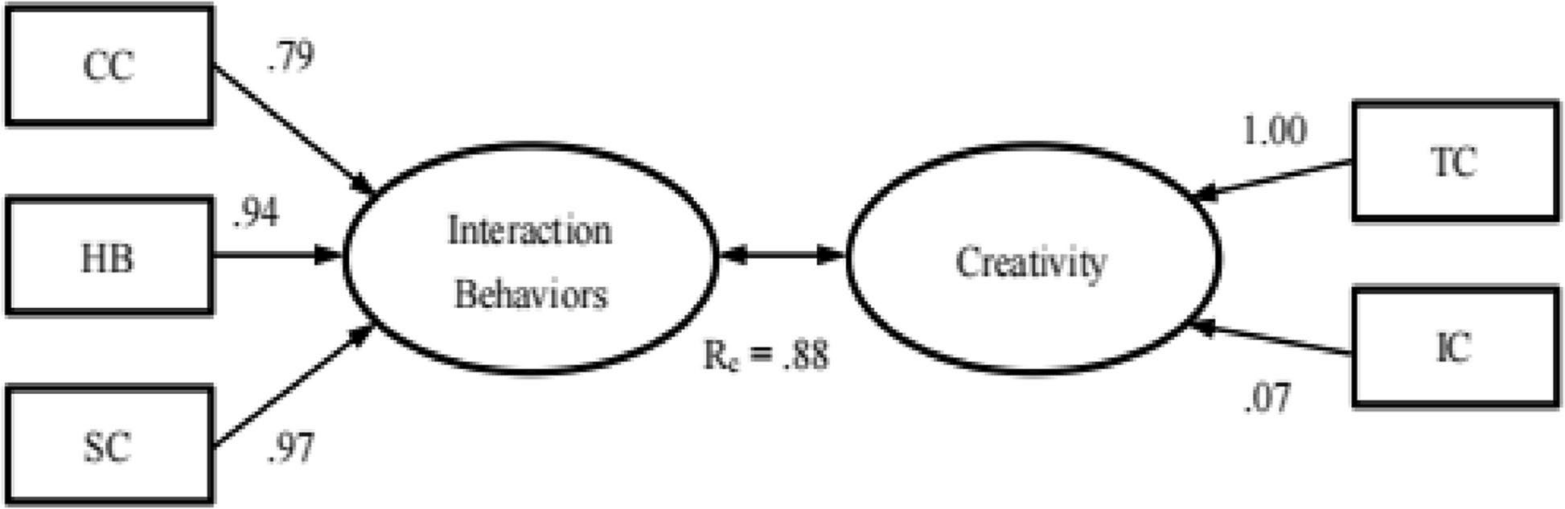
Canonical correlation model for the overall correlation between interaction behavior variables and creativity variables. CC: Constructive Controversy; HB: Helping Behaviors; SC: Spontaneous Communication; TC: Team Creativity; IC: Individual Creativity

Fig. 1 showed the set of independent variables comprised the constructs of constructive controversy, helping behaviors, and spontaneous communication. Importantly, helping behaviors and spontaneous communication were the primary independent variables, with constructive controversy considered secondary. Regarding the set of dependent variables (i.e., creativity constructs), team creativity was the only dependent variable. Given that all structure coefficients for interaction behaviors and team creativity were positive, it indicated that higher scores on interaction behaviors associated with higher team creativity.

### Moderation analysis

Table 4 presented the potential effect of the proportion of females in a team that moderates the relationship between interaction behaviors and team creativity. Model 1 revealed a significant interaction between proportion of females in a team and the total constructive controversy score (Female proportion x CC) (β = 0.43, 95% C.I. [0.07, 0.78], *p* < 0.05), indicating that female gender positively moderated the relationship between constructive controversy and team creativity. In Model 2, the interaction between female proportion and helping behaviors score (Female proportion x HB) was also significant (β = 0.46, 95% C.I. [0.18, 0.74], *p* < 0.01), indicating that female-dominance positively moderated the relationship between helping behaviors and team creativity. However, in Model 3, no statistically significant interaction term between the female proportion and spontaneous communication score (Female proportion x SC) was observed (β = 0.07, 95% C.I. [-0.14, 0.28], *p* = 0.52). This indicated that the gender composition did not act as moderator in the relationship between spontaneous communication and team creativity.

**Table 4:** Regression analysis parameters for examining potential moderating effects of the proportion of females on the relationship between team-level perceptions of each interaction behavior and team creativity scores

Model 1			Model 2			Model 3		
**Variable**	**β**	***SE***	**Variable**	**β**	***SE***	**Variable**	**β**	***SE***
Female proportion (centered)	-1.77^*^ [-3.16, -0.39]	0.70	Female proportion (centered)	-1.88^**^ [-3.00, -0.76]	0.57	Female proportion (centered)	-0.34 [-1.18, 0.48]	-0.34 [-1.18, 0.48]
CC (centered)	0.27 [-0.15, 0.70]	0.22	HB (centered)	0.34^*^ [0.01, 0.66]	0.17	SC (centered)	0.77^***^ [0.50, 1.03]	0.77^***^ [0.50, 1.03]
Female proportion x CC	0.43^*^ [0.07, 0.78]	0.18	Female proportion x HB	0.46^**^ [0.18, 0.74]	0.14	Female proportion x SC	0.07 [-0.14, 0.28]	0.07 [-0.14, 0.28]

*CC* Constructive Controversy, *HB* Helping Behaviors, *SC* Spontaneous Communication, *SE* Standard error, Independent variable, Total interaction behavior score (one per model), Dependent variable, Total team creativity score

^*^p < .05

^**^ p < .01

^***^ p < .001

## Discussion

The canonical correlation analysis confirmed the hypothesis that the set of interaction behaviors would correlate positively with the set of creativity variables. Notably, all three interaction behaviors displayed positive associations with team creativity, while none of the interaction behaviors exhibited a significant relationship with individual creativity. Furthermore, the moderation analysis partially supported that female dominance in nursing teams is a moderator between team interaction and creativity. This indicated that the predominance of female students in the field of nursing may serve as a moderating factor in the relationships between interaction behaviors (constructive controversy and helping behaviors) and team creativity.

Consistent with the literature [37, 38], the students' perceived constructive controversy, helping behaviors, and spontaneous communication were associated with higher perceptions of team creativity in our study. Constructive controversy among the nursing student teams may have allowed team members to be confronted with credible alternative views, the acceptance of which could have helped them generate more imaginative solutions, strategies, and ideas [39]. Additionally, our findings suggest that helping behaviors were important for team creativity, possibly by allowing the students to turn challenges of cooperation, such as diverse ideas or the female-dominated composition of participants, into a resource for innovation [40]. Finally, our findings reinforced the study by McAlpine (2018) which also suggested spontaneous communication is positively associated with team idea generation [41]; using this behavior may have created opportunities for the students to share ideas, generate novel solutions, and engage in collaborative conflict resolution that made room for creativity [42].

Considering the positive impacts of each interaction behavior on team creativity, it is plausible that the combined influence of the three interaction behaviors may have enhanced the creative outcomes of the teams. Our observation that the perceived team interaction behaviors of the students did not correlate with the individual creativity score of each team member. Related research shows that personal personality, personal motivation and work team climate may affect individual creativity [43, 44]. Individual's proactive personality is positively related to individual creativity. Empirical studies have confirmed that proactive personality predicts mutual helping behavior [45], learning behavior [46], and innovation behavior [47]. Additional research would be necessary to ascertain whether team members who actively engaged in the team's interaction behaviors also displayed higher levels of individual creativity.

The majority of students in our study were female. In our investigation of moderating effects, we observed that the predominantly female student composition positively moderated the relationships between team creativity and both constructive controversy and helping behaviors. Considering the gender composition in our study, one might anticipate that the relatively small number of male participants could potentially limit their engagement in helping behaviors [10], consequently affecting the perception of team creativity among students. However, invoking social role theory, which suggests that both women and men exhibit gender-consistent helping behaviors [48], it is conceivable that the team of female predominance may have contributed to creating an environment where male students felt comfortable and supportive expressing helping behaviors. This, in turn, could have led to an enhancement of team creativity, despite the male students' minority representation. Such speculations, coupled with the current lack of literature examining the indirect effects of female dominance on the relationships between interaction behaviors and creativity within the context of nursing education, emphasize the need for further research in this area.

### Limitations

The cross-sectional, single-center study design used in our research, with participants based in Taiwan, necessitates caution when extending our findings to nursing teams in different geographical regions. Furthermore, the reliance on subjective assessments from team members regarding interaction behaviors and team creativity introduces potential limitations. To address these limitations, and enrich the scope of future research, we recommend the inclusion of more objective measures, such as the academic performance of nursing students and standardized creativity assessments evaluated by faculty members. Additionally, the influence of female dominance within nursing settings on interaction behaviors and team creativity reported in this study is only preliminary, warranting further investigation. Future studies are also needed to validate our findings in countries with different nursing education and demographic diversities, to establish the generalizability of the results beyond the specific context of Taiwan, thereby providing a more comprehensive understanding of the interplay between interaction behaviors and team creativity in nursing education.

## Conclusions

The significant positive associations identified in our research suggest several avenues for enhancing the creativity and effectiveness of nursing student teams in their future roles within healthcare teams. First, nursing schools can play a pivotal role by cultivating safe and creatively enriched learning environments. Encouraging interaction behaviors among students within these environments can stimulate creative thinking and problem-solving skills, which are crucial for healthcare professionals. Second, the teaching of specific interaction behaviors, such as helping behaviors and constructive controversy, holds promise for addressing potential conflicts arising from team diversity. By equipping student teams with the skills to effectively engage in these behaviors, nursing educators can foster a cooperative atmosphere that not only reduces conflict but also enhances overall team creativity. Lastly, our findings indicate that nursing student teams characterized by a female-dominated gender distribution may experience amplified positive effects of constructive controversy and helping behaviors on team creativity. This insight could prove valuable for nursing educators responsible for structuring capstone course teams aimed at nurturing creativity among future nurses.

In conclusion, this study demonstrated the importance of nurturing interaction behaviors to foster creative learning environments in nursing education. These efforts can contribute to the development of nursing teams better prepared for the complexities of healthcare teamwork.

### Supplementary Information

Supplementary material 1: Table S1. Sample items from the instruments used to measure nursing students’ perceived team interaction behaviors (TIB).
Table S2. Sample items from the instruments used to measure nursing students’ perceived team creativity (TCr).
Table S3. Sample items from the Torrance Tests of Creative Thinking (TTCT) instruments used to measure nursing students’ individual creativity.

## Data Availability

All the data supporting the study findings are within the manuscript. Additional detailed information and raw data are available from the corresponding author upon reasonable request.
