# Supplementary material for: Team interaction behaviors correlates with team creativity among nursing students: Canonical correlation and moderation analyses
Source: BMC Nurs. 2024 Jul 16;23:484. doi: 10.1186/s12912-024-02158-7 (PMC11251228; doi:10.1186/s12912-024-02158-7)
Supplement: Supplementary file 1 — Supplementary material 1: Table S1. Sample items from the instruments used to measure nursing students’ perceived team interaction behaviors (TIB). Table S2. Sample items from the instruments used to measure nursing students’ perceived team creativity (TCr). Table S3. Sample items from the Torrance Tests of Creative Thinking (TTCT) instruments used to measure nursing students’ individual creativity. [file 12912_2024_2158_MOESM1_ESM.docx]

Appendix

Table S1

Sample items from the instruments used to measure nursing students’ perceived team interaction behaviors (TIB)

| Component | Item |
| --- | --- |
| TIB | During the interdisciplinary course team activities: |
| Constructive controversy | 1. Provide constructive feedback.  2. Respectful of conflicting thoughts and suggestions. |
| Helping behaviors | 1.Offer encouragement if a product fails.  2. Provide constructive suggestions. |
| Spontaneous communication | 1. Willing to exchange different methods.  2. Collaborate with others to stay on schedule. |

Table S2

Sample items from the instruments used to measure nursing students’ perceived team creativity (TCr)

| Component | Item |
| --- | --- |
| TCr | During the interdisciplinary course team activities: |
|  | 1. Team members often propose different kinds of new ideas.  2. Team members create products with originality. |

Table S3

Sample items from the Torrance Tests of Creative Thinking (TTCT) instruments used to measure nursing students’ individual creativity

| Component | Test content | Scoring | Scoring example |
| --- | --- | --- | --- |
| **TTCT-V** | Fill in the 50 blanks in 10 minutes with unusual uses for bamboo chopsticks. |  |  |
| Fluency |  | Total number of answers given by research subjects. | Write 5 uses for 5 scores |
| Flexibility |  | The total number of different categories after all responses are classified.  There are 26 categories in total. |  |
| Originality |  | Originality is calculated separately for each response.  The concept of originality score scoring is normal distribution, originality score is statistically rare numbers.  Scoring：  1.0: the number of responses is more than 5%  2.1: 2%~4.99%  3.2: less than 2% | chopsticks as a tapping or striking tool  book fold/  bookmark  Many pairs of chopsticks joined together to make a book cover. |
| **TTCT-F** | How many humanoid graphics can you draw in 10 minutes? Write the name of the drawing.  There are 57 humanoid graphics of different sizes in total. |  |  |
| Fluency |  | Total number of answers given by research subjects. |  |
| Flexibility |  | The total number of different categories after all responses are classified.  There are 35 categories in total. |  |
| Originality |  | Originality is calculated separately for each response.  The concept of originality score scoring is normal distribution, originality score is statistically rare times.  Scoring：  1.0: the number of responses is more than 5%  2.1: 2%~4.99%  3.2: less than 2% | general person or human being  humanoid graphics drawn as men or women  draw an adult with a child |
| Elaboration |  | Scoring 1 for every appropriate detail attached to the original graphic itself and its surroundings. | The basic shape of a human head is hair, face and facial features. If a human head is drawn with only these, the elaboration scoring will be 0. If one more hair accessory is drawn, the elaboration scoring will be 1. |
